# The Mixed Effect of Role Overload on Extra-Role Performance: The Mediation Role of Behavioral Inhibition System/Behavioral Activation System Responses

**DOI:** 10.3389/fpsyg.2021.748732

**Published:** 2021-11-30

**Authors:** Bo Huang, Lina Ma, Wei Xia

**Affiliations:** ^1^School of Labor and Human Resources, Renmin University of China, Beijing, China; ^2^School of Education Science, Sichuan Normal University, Chengdu, China; ^3^School of Preschool and Elementary Education, China West Normal University, Nanchong, China

**Keywords:** role overload, hindrance stressors, challenge stressors, withdrawal, job crafting, extra-role performance, voice, leader-member exchange

## Abstract

The findings of existing studies of how role overload affects employees’ performance in organizations have been mixed and controversial. We draw on the hindrance–challenge framework to suggest that role overload contains both hindrance and challenge stressor components. We integrate this theory with the behavioral inhibition and behavioral activation systems (BIS and BAS) perspective to develop hypotheses about the effects of role overload on employees’ extra-role performance (voice). We suggest that although role overload is positively associated with withdrawal (a prototypical response of the BIS system) and ultimately negatively influences extra-role performance, it can also trigger job crafting (a prototypical response of the BAS system) and is, consequently, positively associated with extra-role performance. We further posit that the strength of these indirect effects is moderated by the quality of leader–member exchange (LMX). To support these hypotheses, we conducted a time-lagged study of 450 full-time pre-school teachers from various Chinese kindergartens. As hypothesized, we found that withdrawal and job crafting mediated the relationship between role overload and extra-role performance. Further, LMX strengthens the positive relationship between role overload and job crafting. Taken together, our results suggest that role overload can be a mixed stressor that activates both negative and positive behaviors, thus ultimately having an impact on extra-role performance.

## Introduction

Job stressors have long been an important topic in organizational and industrial psychology (Cooper et al., 2003). Increasingly business competition and the recent sanitary crisis (the COVID-19 pandemic) have led to an increase in stressors such as workload, job insecurity, and job instability (Bliese et al., 2017; Nemteanu and Dabija, 2021; Nemteanu et al., 2021). In addition, technological advances have led employees to be exposed to workplace stressors more frequently (Monni et al., 2020). For example, the development of mobile communication technology makes employees have to work anywhere, anytime (Colbert et al., 2016), and organizations are increasingly using the latest technology to monitor employee performance (Harrower, 2019; Kassick, 2019; Pera, 2019; Wingard, 2019), which increases workers’ perceptions of stress (Barley et al., 2011). Indeed, a report revealed that around 64% of respondents cited “work” as a major source of stress (American Psychological Association, 2019). The purpose of this paper is to explore in the context work-related stress grew and developed over the century, whether and how job stressors affect employees’ extra-role performance.

Broadly defined, job stressors refer to situations in which job-related factors require an employee to make an adaptive response of some kind (Kahn and Byosiere, 1992; Sacramento et al., 2013). Stressors are often said to be a critical factor that has a significant impact on both employees and organizations (Lepine et al., 2005; Podsakoff et al., 2007). Role overload is one of the major work-related stressors (Cooper, 1987) and refers to situations in which employees perceive themselves as having too many responsibilities or activities to take on given the resources available to them (e.g., time, ability). There are mixed findings regarding the relationship between role overload and work-related outcomes. For example, some studies found that role overload is negatively related to organizational commitment (Fisher, 2014) and organizational citizenship behaviors (OCBs) (Carlson et al., 2019) and positively related to anxiety (Caplan and Jones, 1975) and work–family conflict (Carlson et al., 2019). However, some other studies showed the opposite and found that role overload is positively related to job satisfaction (Beehr et al., 2001), creativity (Ohly and Fritz, 2010), loyalty (Boswell et al., 2004), motivation to learn (LePine et al., 2004) and negatively related to absenteeism (Dwyer and Ganster, 1991), withdrawal (Boswell et al., 2004), and turnover intention (Beehr et al., 2001; Boswell et al., 2004). Moreover, based on meta-analysis results, role overload is a weak predictor of poor job outcome indicators (Örtqvist and Wincent, 2006; Eatough et al., 2011; Bowlin et al., 2015). Such inconsistency between research findings may indicate the limitations of the current theoretical framework.

The hindrance–challenge framework of stressors is currently the most popular theoretical ground for understanding work stressors (Cavanaugh et al., 2000). Moreover, the potentially most important outcome for employees is their extra-role performance (Griffin et al., 2007). In particular, OCBs, which are defined as discretionary behaviors by employees that are considered to directly advance the effectiveness of an organization (Organ, 1988), have received the most attention. Briefly stated, the hindrance–challenge framework suggests that stressors are composed of two dimensions, i.e., hindrance and challenge stressors (LePine et al., 2004). Both types of stressors are associated with strains; nevertheless, hindrance stressors are harmful, whereas challenge stressors have the potential to lead to employees’ gain and growth, and are thus beneficial to their performance (LePine et al., 2004). The hindrance–challenge framework has been applied broadly to explore the relationships between work stressors and job performance (Zhang et al., 2014). However, due to the inconsistent research findings noted above, a consensus has yet to be reached on the relationship between role overload and critical workplace outcomes at work, and accounting for this inconsistency is crucial for theoretical and practical reasons. Moreover, as suggested by Zhang et al. (2014), it would be valuable to propose an explanation of stressor effects based on a different theoretical perspective. Hence, our current study integrates the hindrance–challenge framework with the behavioral inhibition system/behavioral activation system (BIS/BAS) perspective (Gray, 1990; Carver, 2006) to propose that role overload contains components both of hindrance and challenge stressors simultaneously for employees, and we argue that role overload may trigger withdrawal and job crafting (the prototypical BIS and BAS responses, respectively), eventually having either negative or positive influences on OCBs, which are the most important extra-role performance for the organization.

Understanding the relationship between role overload and OCBs is invaluable for the following two reasons. First, as suggested by Örtqvist and Wincent (2006), different role stressors produce diverse results; it is, therefore, useful to focus on each role stressor individually. Second, the nature of the relationship between role overload and OCBs is more controversial than other role stressors. To be specific, some studies (e.g., Zhang and Xie, 2017; Carlson et al., 2019) supported the hypothesis that role overload is negatively related to OCBs. Some studies (e.g., Pooja et al., 2016; Montani et al., 2017), however, did not support this relationship, and even found the opposite, that is, role overload is positively related to OCBs (e.g., Montani et al., 2017). Evidence from meta-analysis also indicated that unlike role ambiguity and role conflict (two other major stressors), the relationship between role overload and OCBs is not significant (Eatough et al., 2011). On account of these incompatible empirical findings, Eatough et al. (2011, p. 626) emphasized that “role overload, however, had a more complex relationship with OCB.” For those reasons, we hope this study will provide valuable evidence for the literature on role overload and OCBs.

**FIGURE 1:**
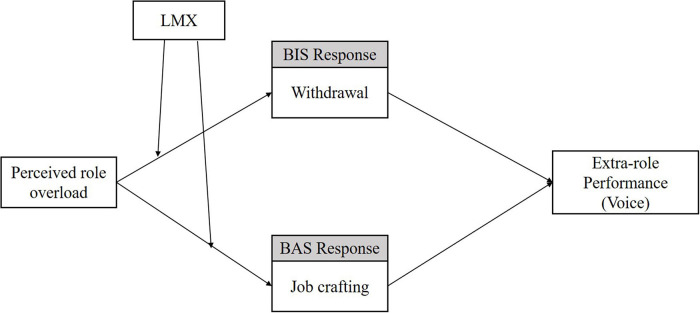
Theoretical model of the current research.

We further explored whether the linkage between role overload and OCBs is moderated by leader–member exchange (LMX) (Graen and Uhl-Bien, 1995). The theory of leadership and LMX suggests that leaders can influence how employees interpret job characteristics (Piccolo and Colquitt, 2006). Moreover, LMX can play a role as a resource for employees to buffer the negative effects of their job demands (Demerouti et al., 2011; Xia et al., 2020). Hence, we argue that the higher the LMX, the less likely role overload be perceived by employees as a hindrance stressor and more likely perceived as a challenge stressor. Therefore a high-LMX is more likely to trigger the BAS response job crafting, which is ultimately positively associated with OCBs. Thus, we theorize that the effect of role overload on BIS/BAS behavioral responses will be moderated by LMX and ultimately be associated with employees’ OCBs. Figure 1 illustrates our theoretical model.

The current study makes three primary contributions to the extant literature. First, we follow the recommendation by Zhang et al. (2014) to introduce a new perspective that may further explain the effect of role stressors on employees. By integrating the hindrance–challenge stressors framework and the BIS/BAS perspective, we identify the BIS/BAS responses withdrawal and job crafting as a key mechanism linking role overload to OCBs; we provide a possible solution to the contradictory findings on the impact of role overload on OCBs (Eatough et al., 2011). Second, in contrast to the vast majority of studies suggesting that role overload should either be treated as a hindrance stressor (Crawford et al., 2010; Tang and Vandenberghe, 2019) or a challenge stressor (LePine et al., 2004; Montani et al., 2017), our study is in accordance with the idea of Eatough et al. (2011) that role overload is a complex role stressor that has components of both hindrance and challenge stressors. To the best of our knowledge, prior studies have not yet tested this mixing effect of role overload on OCBs in one analytic model, as they have typically explored the effect of role overload in separate models by different samples. Unfortunately, this cannot examine the possible mixing effect of role overload. Hence, our study examines the two different mechanisms by which role overload may be triggered in one model. This analysis, therefore, allows us to provide evidence about the duality of role overload. Third, we examine the potential moderating effect of LMX in the role overload—OCBs relationships, which may expand our understanding of the contextual factors that can influence the effect of role overload on OCBs.

In the sections that follow, we review relevant work from the stressor’s literature. We then elaborate our theoretical orientation and offer hypotheses that speak to the mediating effects of the two prototypical behavioral responses of BIS and BAS (withdrawal and job crafting) and the moderating effects of LMX. Finally, we report on tests of our hypotheses in a multi-wave field study of kindergarten teachers.

## Theoretical Background and Hypotheses

### Role Overload as a Stressor

Researchers have identified five categories of stressors in the workplace (Cartwright and Cooper, 1997): the internal factors of the job itself (e.g., noise), role stressors (e.g., role conflict), relationships (e.g., abusive supervision), career issues (e.g., job insecurity), and organizational factors (e.g., political climate). Among these job-related stressors, role stressors are the most regularly explored (Tubre and Collins, 2000). The pioneering investigation by Kahn is often regarded as the starting point for role stressors research (Cooper et al., 2003). According to Katz and Kahn (1978), role stressors arise in two main ways: role ambiguity and role conflict. Role ambiguity refers to a lack of clarity and certainty regarding an employee’s job responsibilities; as a result, employees do not know what is expected of them (Katz and Kahn, 1978). For instance, a kindergarten teacher might experience role ambiguity if the supervisor asked them to reduce the stunting rate of children to 0.5% in the next 3 years but did not illustrate the particular tasks that needed to be done to achieve the goal (e.g., educating parents and kids about food safety, optimizing the physical education curriculum). Role conflict refers to the experience of disparate or contradictory anticipations and demands that make it difficult to perform work tasks (Katz and Kahn, 1978). For example, a kindergarten teacher is not only required to teach children some skills in order to fulfill their role as teacher but also needs to pay attention to the safety and physical needs of the children to fulfill their role as a parent.

Researchers have found that role overload is also a major predictor of work-related stressors (Cooper, 1987). Role overload refers to situations in which employees perceive themselves as having too many responsibilities or activities to take on given the resources (e.g., time, ability) available to them (Rizzo and House, 1970). For instance, in addition to their daily teaching activities, a kindergarten teacher needs to undertake various kinds of administrative work and needs to work overtime frequently. Studies of role stressors have therefore adopted this trilogy of workplace stressors (Gilboa et al., 2008; Eatough et al., 2011). Studies have demonstrated a consistently harmful relationship between role ambiguity and role conflict and many important personal and organizational outcomes (Tubre and Collins, 2000). As a result, role stressors are typically considered to have a negative impact on employees (Fisher, 2014).

The existing debate about job stressors concentrates on role overload, as we have mentioned above. Due to the inconsistency of the research conclusions, researchers have tried to put forward theories to understand workplace stressors. On the one hand, the inconsistent findings are explained as the degree of stress, suggesting that an optimal stress range exists; neither too much stress nor too little stress is good for performance (Shultz et al., 2010). However, this suggestion has received limited support (Fay and Sonnentag, 2002; LePine et al., 2004). On the other hand, the inconsistent findings are explained by the nature of the stressors (Jex, 1998). Depending on whether stressors can bring growth and gains, researchers differentiate between stressors as hindrance and challenge stressors (Cavanaugh et al., 2000). Hindrance stressors are those assessed as being potentially threatening to personal growth and gain, which can trigger negative emotions and aversive work outcomes (Zhou et al., 2019). In contrast, challenge stressors are those assessed as being potentially beneficial for personal growth and gain. Challenge stressors can trigger positive emotions and desired work outcomes (Yang and Li, 2021). Depending on the hindrance–challenge framework, studies indeed found that hindrance stressors have a negative and challenge stressors a positive relationship with important occupational criteria (Cavanaugh et al., 2000; Boswell et al., 2004; Lepine et al., 2005).

The hindrance–challenge framework provides important insights into our understanding of occupational stressors. However, a consensus has yet to be reached. The debate centers on whether role overload is a hindrance stressor or a challenge stressor. For example, some researchers have suggested that role overload is a hindrance stressor and provide evidence about the negative effects of role overload (Lepine et al., 2005; Podsakoff et al., 2007; Crawford et al., 2010; Tang and Vandenberghe, 2019). Some researchers believe that role overload is a challenge stressor; they also contribute evidence about the positive effect of role overload (Cavanaugh et al., 2000; Fay and Sonnentag, 2002; Boswell et al., 2004; LePine et al., 2004; Ohly and Fritz, 2010; Montani et al., 2017). In addition, some researchers hold a contingent view that role overload is a challenge stressor under certain conditions (Gilboa et al., 2008; Solberg and Wong, 2016) or that role overload is a hindrance stressor for some outcomes but a challenge for others (Carlson et al., 2019). It is not difficult to infer from the above arguments that the situation of role overload is complex. As Eatough, Chang, Miloslavic, and Johnson have stated, “it [role overload] may have a more complex relationship… compared with the other role stressors” (Eatough et al., 2011, p. 620). Due to the complexity and controversial nature of the research findings, it is beneficial to re-examine role overload from a different perspective. In our study, we integrated the hindrance–challenge framework and the perspective of BIS and BAS to examine the relationship between role overload and extra-role performance. Next, we will briefly introduce the core ideas of the BIS/BAS framework.

### Behavioral Inhibition System and Behavioral Activation System

That humans seek pleasure and try to avoid pain is the core idea of various psychological theories (Higgins, 1997). Those theories seek to understand how individuals respond to environmental cues through approach or avoidant behaviors (Monni et al., 2020). The foundational ideas of the BIS/BAS are that human behaviors are controlled by two biological self-regulatory systems; the two systems guide individuals’ goal selection and striving process (Carver and Scheier, 1982; Gray, 1990). The BIS and BAS are functionally independent and have different neural substrates, and have different effects on actions (Carver, 2006).

The BIS regulates aversive motivation and controls the experience of anxiety and sensitivity to cues of penalty or no reward (Carver, 2006; Elliot, 2006). Once BIS is triggered by punishment-relevant or potential harmful cues in the environment, it motivates individuals away from those negative or painful situations and inhibits behaviors toward the target (Carver and White, 1994). In contrast, the BAS regulates appetitive motivation and is sensitive to information regarding rewards (Carver and White, 1994; Carver, 2006). Once BAS is triggered by potential positive cues, it motivates individuals toward goals or potential rewards and opportunities (Carver, 2006; Elliot, 2006). The BIS/BAS perspective is often used as a theoretical lens for the study of biology and neuroscience (Corr and McNaughton, 2012) and of individual difference (Li et al., 2015) and serves as a theoretical foundation for exploring behavioral responses when faced with threatening or rewarding stimuli (Sherf et al., 2021). In our study, we adopt the last way the BIS/BAS perspective is applied and argue that role overload may serve as an environmental cue to activate the BIS and BAS regulation systems.

Researchers have proposed that humans are surrounded by various reward and punishment stimuli or situations that act as extrinsic motivational events (Monni et al., 2020). People make their own evaluations of these surrounding stimuli, so different people or an individual in different circumstances can perceive the same stimuli or situations either positively or negatively (Gary, 1982; Monni et al., 2020). In other words, in different individuals’ eyes, the same stimulus can have rewarding or threatening characteristics (Monni et al., 2020). According to this reasoning, we argue that role overload may be perceived as a hindrance or a challenge stressor by different employees.

On the one hand, role overload describes situations in which employees perceives themselves as having been assigned too much work to be handled effectively (Sutherland and Cooper, 2000). Role overload situations represent an uncontrollable demand on employees that goes beyond their capabilities or coping resources (Rizzo and House, 1970; Gilboa et al., 2008). In such a situation, the quality of the employee’s work may be interfered with, which, in turn, may detract from their performance evaluation result and bonus allocation. Thus, an employee may perceive role overload as a threat and hindrance to their job performance. In addition, role overload leads to a degree of uncertainty about people’s ability to get the job done with limited time and energy (Crawford et al., 2010). In this way, the possible threat of losing valuable work-related resources is amplified (Hobfoll, 1989). Furthermore, role overload may hamper employees’ need for autonomy, thereby negatively influencing work outcomes (Tang and Vandenberghe, 2019). For example, too many activities being assigned to a kindergarten teacher may interfere with the quality of their teaching, which is the core of a teacher’s success at work. For these reasons, employees may perceive role overload as a hindrance stressor (Tang and Vandenberghe, 2019). In this vein, the BIS is engaged by signals of punishment, no reward, or disagreeable threatening situations—all of which are characteristics of hindrance stressors (Gray, 1981). Therefore, role overload as a hindrance stressor in this situation may serve as an environmental cue to activate the BIS regulations.

On the other hand, role overload may also describe situations in which employees perceive themselves as having been given more responsibilities and work challenges (Montani and Dagenais-Desmarais, 2018). Such situations may imply that more career development, chances to demonstrate ability, skill learning, and rewarding opportunities are possible for the employees (LePine et al., 2004). For instance, a kindergarten teacher is assigned new teaching tasks and has to prepare a Children’s Day party within a specified time. The teacher is likely to experience role overload, but they may also perceive that such a task arrangement may allow them to learn new teaching and leadership skills. This perception may motivate the teacher to devote more energy to completing the task. Indeed, Boswell et al. (2004) found that employees who experience greater work responsibilities and workloads may feel more challenged at work, which positively influences their job attitudes, retention intention, and work behaviors. Role overload may make employees feel responsible for their work and thus gain self-competency. Psychological theory has suggested that the need for competency is a basic human need (Deci and Ryan, 2000). Hence, some researchers have suggested that workload is essential for employees. For example, Chiu et al. (2015) found that role conflict and role ambiguity have a positive relationship with deviant behaviors, whereas role overload has a negative one. For these reasons, employees may also perceive role overload as a challenge stressor (Cavanaugh et al., 2000; Sacramento et al., 2013; Montani et al., 2017). Under these circumstances, the BAS is engaged by cues of potential reward, personal growth, and motivating situations—all of which are features of challenge stressors (Carver, 2006). Hence, role overload as a challenge stressor in this situation may serve as an environmental cue to activate the BAS regulations.

### Withdrawal as a Behavioral Inhibition System Response and Job Crafting as a Behavioral Activation System Response

As we have noted, the BIS and BAS are neurobiological systems associated with different behaviors (Sherf et al., 2021). We suggest that withdrawal stands for a prototypical response to BIS regulations in the workplace, whereas job crafting represents a typical reaction to BAS. Conceptually, withdrawal is defined as a “set of behaviors dissatisfied individuals enact to avoid the work situation” (Hanisch and Hulin, 1990, p. 63). Hanisch and Hulin (1990) suggested that employees’ fundamental attitudes and affect with respect to their work roles are reflected by withdrawal. Inconsistent with withdrawal, job crafting refers to proactive changes made by employees in order to balance job resources and demands based on their own preferences and needs (Tims et al., 2012).

When justifying whether a behavior is a prototypical response of BAS or BIS, one usually starts from two aspects: the goals and the actions (Elliot, 2006; Sherf et al., 2021). For goals, withdrawal has the objective of avoiding one’s job tasks while maintaining organizational membership (Hanisch and Hulin, 1990). Researchers have suggested that employees who experience disagreeable characteristics or negative events at work may withdraw from these unfavorable work conditions (e.g., sexual harassment, pay inequity) (Kathy et al., 1998). In this way, the goal of withdrawal—avoiding negative stimuli or work conditions—fits with the objective of the BIS, as “escaping from and rectifying existing negative situations” (Elliot, 2006, p. 112) and job crafting is an approach-oriented behavior with the objective of varying tasks or job characteristics proactively (Tims et al., 2012). Thus, as a proactive behavior, the goal of job crafting—“to set and attain goals” (Demerouti, 2014, p. 240)—fits well with the objective of the BAS: “getting something positive that is currently absent” (Elliot, 2006, p. 112).

For actions, withdrawal involves a variety of inhibition-oriented acts, such as lateness, absenteeism, and deliberately lowering work effort (Hanisch and Hulin, 1990), which correspond with the behaviors resulting from the BIS—“action away from (i.e., avoiding, preventing, or inhibiting) potential punishment or harm to the self” (Sherf et al., 2021, p. 116). In this way, acts of withdrawal may have a goodness of fit with BIS. In contrast, job crafting is a self-initiated and change-oriented behavior, such as seeking to learn something new at work (Tims et al., 2012). Thus, the action of job crafting may also correspond with the behaviors resulting from the BAS: “action toward (i.e., approaching, seeking, achieving) potential opportunities and reward to the self” (Sherf et al., 2021, p. 116).

These statements allow us to advance the hypothesis that role overload triggers withdrawal—which represents a prototypical BIS response—as well as job crafting, a prototypical BAS reaction. In fact, the idea of the dual nature of role overload is not new. Eatough et al. (2011) suggested that “role overload has both strong hindrance and challenge components” (p. 620). Similarly, Gilboa et al. (2008) also suggested that role overload may represent both a challenge and a hindrance to employees. Empirical studies give some support for this hypothesis. For instance, Rodell and Judge (2009) found that role overload has a positive effect on withdrawal. Rudolph et al. (2017) found that workload is positively associated with job crafting. Thus, based on relevant theory and research, we hypothesize:

H1a: Role overload is positively associated with withdrawal.

H1b: Role overload is positively associated with job crafting.

### Role Overload and Extra-Role Performance

Performance is regarded as a multi-dimensional construct (Campbell, 1990). In addition to in-role performance, extra-role performance also has a significant impact on organizational success (MacKenzie et al., 1998). Among the multiple forms of extra-role performance, OCBs have received the most attention (MacKenzie et al., 1998). Organ (1988) defines OCBs as discretionary behaviors by employees that are believed to facilitate the effectiveness of an organization. OCBs can be divided into two categories according to the objects of action (Organ and Konovsky, 1989): OCB-I, OCBs that benefit individuals (e.g., helping), and OCB-O, OCBs that benefit the organization (e.g., work overtime). In our current study, we focus on the relationship between role overload and OCB-Os. Specifically, we examine the link between role overload and one of the most important OCBs, promotive voice, which is defined as the expression of new ideas or suggestions by employees to improve the overall performance of their organization (Liang et al., 2012).

It is necessary to illustrate why we focus on the relationship between role overload and promotive voice. First, role overload is intrinsically relevant to how tasks are organized within the organization (Tang and Vandenberghe, 2019). Therefore, employees are more inclined to attribute strain to the organization in which they work than to the other employees in the same organization (Siegrist, 1996). In addition, OCB-I represents affiliative promotive behavior that tends to preserve the relationship with other employees, and OCB-O represents challenging promotive behaviors that might damage relationships with others (Van Dyne and LePine, 1998). Thus, as suggested by Eatough et al. (2011), the organization-directed OCB-O, rather than the people-directed OCB-I, may be more likely to be influenced by role overload. Second, we concentrate on promotive voice rather than other forms of voice because promotive voice behavior is a change- and approach-oriented behavior (Liu et al., 2015), which highlights better work practices and opportunities to improve performance. Therefore, in alignment with prior studies (Aryee et al., 2017), we also conceptualize promotive voice as a behavior that involves challenging the *status quo* and has both benefits and risks for the actors. Based on this, the feature of role overload—which contains hindrance and challenge components (Eatough et al., 2011)—may be reflected by promotive voice behavior, which contains both risks and benefits. Based on the above reasons, our study focuses on the link between role overload and promotive voice.

Based on the BIS/BAS perspective, BIS regulation may negatively affect employees’ performance, as BIS is intrinsically aversive and employees’ energy or intrinsic work motivation may be reduced (Carver, 2006; Elliot, 2006; Sherf et al., 2021). Correspondingly, the representative behavior of BIS regulation, withdrawal, may also weaken extra-role performance. For this reason, we expect that role overload may have negative effects on extra-role performance through withdrawal. Empirical evidence supports the idea that employees who exhibit withdrawal are likely to experience performance reduction (Maslach and Leiter, 2008).

We also expect that role overload may have positive effects on extra-role performance through job crafting. Unlike the BIS, the BAS is related to striving for and obtaining rewards and opportunities (Carver, 2006; Sherf et al., 2021). Correspondingly, the representative behavior of BAS regulation, job crafting, may strengthen extra-role performance. Likewise, studies support the idea that employees who exhibit job crafting show higher performance (Lin et al., 2017). Thus, based on this rationale from the BIS/BAS perspective, as well as evidence from the withdrawal and job crafting literature, we anticipate that:

H2a: Mediated through withdrawal, role overload is negatively associated with extra-role performance.

H2b: Mediated through job crafting, role overload is positively associated with extra-role performance.

### The Moderating Role of Leader–Member Exchange

Thus far, Hypotheses 1–4 provide an account of the effects of role overload on employees’ extra-role performance. We have hypothesized that withdrawal and job crafting, which represent the prototypical behavioral responses of BIS and BAS, respectively, serve as a key intervening mechanism that explains the relationship between role overload and extra-role performance. In this section, we explore the question of when role overload is more or less harmful or beneficial for employees. This discussion is grounded in the notion that leaders play an important role in employees’ interpretation of their work environment (Yam et al., 2018). Piccolo and Colquitt (2006) suggested that leaders can define the “reality” in which followers work, thus influencing how followers interpret their job’s characteristics. In the occupational stressors literature, Zhang et al. (2014) found that transformational leadership has a significant impact on the association between stressors (both challenge and hindrance stressors) and perceptions of the environment.

Following the LMX theory, a leader can have diverse relationships with the members of their work units (Graen and Uhl-Bien, 1995); some are in-group members who have special, high-quality exchange relationships with the leader (Graen and Uhl-Bien, 1995). Compared to low-quality LMX workers (out-group members), an employee in a good LMX relationship (in-group member) will benefit more from the leaders (Dienesch and Liden, 1986). Grounded in this notion, we suggest that LMX, which serves as an additional resource and contextual information for followers to interpret the character of role overload, can attenuate the negative effects of role overload through withdrawal while exacerbating the positive effects through job crafting on employees’ extra-role performance.

On the one hand, a good relationship with one’s supervisor can serve as a job resource; for example, one may receive more support, encouragement, and concern from the leader (Liden and Graen, 1980). Followers in high-LMX relationships receive more access to information and decision-making opportunities and should perceive themselves as being more empowered by their leader (Prof and Yagil, 2005), all of which should increase employees’ competence consciousness, perception of self-determination, and self-efficacy (Dulebohn et al., 2012). As a result, with the resources provided by LMX, employees are more likely to view role overload as a challenge stressor. On the other hand, having a good relationship with a supervisor may promote employees’ expectations about the possibility of being rewarded for their hard work, thus affecting employees’ judgment about the characteristics of stressors. More specifically, when employees have been exposed to hindrance stressors, they may find it difficult to meet these demands and thus are less likely to receive valuable rewards (Zhang et al., 2014; Montani et al., 2017). Conversely, addressing challenging stressors can make employees feel rewarded (e.g., personal growth) (Podsakoff et al., 2007; Montani et al., 2017). Thus, based on these theories and evidence, it is reasonable to infer that the key for an individual to experience role overload as a hindrance or challenge stressor may largely depend on employees’ judgment about the possibility of acquiring reward after effort investing.

Studies have revealed that followers in high-LMX relationships have more opportunities to gain rewards for their contributions (Dienesch and Liden, 1986; Roch and Shanock, 2006). Meta-analysis results also indicate that high-LMX is positively associated with satisfaction with pay (Dulebohn et al., 2012). As a result, we suggest that due to the resource support from the leader, higher perception of competence, as well as the possibility of being rewarded, the in-group members are more likely to view stressors as less threatening and to interpret role overload as a challenge rather than a hindrance stressor. Indeed, Nelson et al. (1998) found that employees with high-LMX are less likely to perceive role conflict or role ambiguity (both of which are hindrance stressors). Therefore, the negative indirect effects of role overload on extra-role performance via withdrawal should be mitigated, and the positive indirect effects of role overload on extra-role performance through job crafting should be enhanced. Thus, we propose the following hypothesis:

H3a: The negative indirect effect of role overload on extra-role performance via withdrawal is moderated by LMX such that the relationship is weaker when LMX is higher but stronger when LMX is lower.

H3b: The positive indirect effect of role overload on extra-role performance via job crafting is moderated by LMX such that the relationship is stronger when LMX is higher but weaker when LMX is lower.

## Materials and Methods

### Sample and Data Collection

In order to explore how role overload influences employees’ extra-role performance in the context of increasingly job stressors, the author adopted a quantitative study on Chinese employees. The sample of our study is composed of full-time kindergarten teachers in China. Studies have indicated that, due to the multiple responsibilities early childhood teachers need to take on, early childhood education is a highly stressful occupation (Gu et al., 2020).

Data were collected through a web-based survey as part of a larger study. The study participants consisted of 450 full-time kindergarten teachers from 43 cities in 17 provinces in China. A two-wave survey with a 1-month time lag was used to reduce common method bias (Podsakoff et al., 2003). The teachers who participated in the survey were informed beforehand of the anonymity and confidentiality of the survey, and all questionnaires were conducted online. Each kindergarten teacher was compensated with RMB 15 (about US $2) per survey to encourage participation. At Time 1, we contacted 1,500 full-time kindergarten teachers and asked them to report their perceived role overload, perceived LMX, and demographic information; a total of 660 kindergarten teachers completed the questionnaires, yielding a 44% response rate. At Time 2, kindergarten teachers who completed the first wave survey were asked again to report their withdrawal behavior, job crafting, and extra-role performance (voice). A total of 450 valid questionnaires were obtained. Of these kindergarten teachers (*N* = 450), 447 were women (99.3%). The average tenure was 7.56 years (SD = 9.06), the average age was 30.40 years (SD = 8.27). In terms of education, 417 (92.7%) had a bachelor’s degree or above.

### Measurement Instruments

We used the five-item role overload scale developed by Peterson et al. (1995) to measure role overload. This scale has demonstrated good validity in the context of China (Zhang and Xie, 2017). The items were scored on a five-point Likert scale ranging from 1 (strongly disagree) to 5 (strongly agree), a typical item being “My workload is too heavy.” The Cronbach’s alpha for this scale was 0.93.

The kindergarten teachers’ withdrawal behavior was measured using two subscales of the On-the-Job behavior scale developed by Lehman and Simpson (1992). Four dimensions—positive work behavior, psychological withdrawal behavior, physical withdrawal behavior, and oppositional work behavior—are included in this scale. In our study, we chose the psychological withdrawal dimension (eight items, an example item is “spending work time on personal matters”), as well as the physical withdrawal dimension (four items, an example item is “having fallen asleep at work”) to measure withdrawal behavior. Good validity in a Chinese sample was demonstrated (Yuan et al., 2021). The items were scored on a seven-point Likert scale ranging from 1 (never) to 7 (very often). The Cronbach’s alpha for these items was 0.88.

We measured job crafting by adapting the 21-item Job Crafting Scale (Tims et al., 2012). This scale includes four dimensions: increasing structural resources (five items, e.g., “I try to develop my capabilities”), decreasing hindering job demands (six items, e.g., “I organize my work in such a way as to make sure that I do not have to concentrate for too long a period at once”), increasing social job resources (five items, e.g., “I ask my supervisor to coach me”), increasing challenging job demands (five items, e.g., “I try to make my work more challenging by examining the underlying relationships between aspects of my job”). This scale has demonstrated good validity in the context of China (Guan and Frenkel, 2019). The kindergarten teachers were asked to respond on a five-point scale ranging from 1 (never) to 5 (often). The mean Cronbach’s alpha for these items was 0.80.

In line with previous research (Liu et al., 2015; Aryee et al., 2017), we used the Liang et al. (2012) five-item promotive voice scale to measure voice behavior. This scale has demonstrated good validity in the context of China (Liang et al., 2012). The items were scored on a five-point Likert scale ranging from 1 (strongly disagree) to 5 (strongly agree), a typical item being “raising suggestions to improve the kindergarten’s working procedure.” The scale’s alpha reliability in this study is 0.91.

Leader–member exchange was assessed using the seven-item scale developed by Scandura and Graen (1984). This scale has been validated in China (Zhang et al., 2015). The respondents were asked, for example, if they agreed with the statement “I have an effective working relationship with my supervisor.” The items were scored on a six-point Likert scale ranging from 1 (strongly disagree) to 6 (strongly agree). The Cronbach’s alpha for this scale was 0.87.

We also controlled for the effects of participant age, tenure, education, and income to exclude possible confounding factors when predicting performance (Eldor and Harpaz, 2016).

## Results

### Variable Discriminant Validity Test

SPSS 26.0 and AMOS 26.0 were used for the statistical analysis in the current study. To examine whether role overload, withdrawal, job crafting, and voice were distinct constructs, we conducted a confirmatory factor analysis (CFA). As Table 1 shows, the fit-indices of the four-factor model were good, indicating the independence of these constructs.

**TABLE 1:** Confirmatory factor analysis (CFA) of measurement models.

Model	x^2^	df	x^2^/df	CFI	TLI	SRMR	RMSEA
Four-factor model	212.979	98	2.173	0.972	0.966	0.036	0.051
Three-factor model	477.412	101	4.727	0.908	0.891	0.090	0.091
Two-factor model	834.540	103	8.102	0.821	0.792	0.140	0.126
One-factor model	2276.217	104	21.887	0.469	0.387	0.226	0.216

*A four-factor model composed of role overload, withdrawal, job crafting, voice. A three-factor model with role overload, withdrawal combined. A two-factor model with role overload, withdrawal, and job crafting combined. A one-factor model with role overload, withdrawal, job crafting, and voice loaded onto a single factor.*

### Descriptive Statistics and Correlation Analysis

Table 2 displays the means, standard deviations, correlations, and internal consistency reliability estimates for all study variables. As shown in Table 2, each study variable has an acceptable degree of internal consistency reliability. The correlations among the study variables are generally consistent with expectations. For instance, a positive correlation was found between employees’ perception of role overload and withdrawal (*r* = 0.29, *p* < 0.01) as well as job crafting (*r* = 0.14, *p* < 0.01), providing preliminary support for the notion that role overload contains hindrance and challenge components (Eatough et al., 2011). Moreover, in line with prior research (Pooja et al., 2016), the correlation be-tween employees’ perception of role overload and voice is not significant (*r* = −0.05), further indicating the complexity of role overload (Eatough et al., 2011). It can also be seen that there is a significant correlation between withdrawal/job crafting and voice.

**TABLE 2:** Means, standard deviations, and correlations among the variables.

	*M*	SD	1	2	3	4	5	6	7	8	9
1. Age	30.40	8.27	–								
2. Education	2.59	0.64	−0.11*	–							
3. Income	7.56	9.06	0.86**	0.00	–						
4. Tenure	3.68	1.64	0.30**	0.48**	0.37**	–					
5. Role Overload	3.40	0.98	0.07	0.20**	0.07	0.13**	(0.93)				
6. Withdrawal	2.05	0.78	−0.22**	0.26**	−0.13**	0.03	0.29**	(0.88)			
7. Job Crafting	3.52	0.41	0.20**	0.03	0.17**	0.06	0.14**	−0.10*	(0.80)		
8. Voice	3.78	0.55	0.23**	−0.10*	0.20**	0.04	–0.05	−0.32**	0.31**	(0.91)	
9. LMX	3.88	0.87	0.23**	−0.15**	0.16**	–0.06	−0.13**	−0.34**	0.34**	0.45**	(0.87)

n = 466; **p < 0.01, *p < 0.05. Cronbach’s alpha is in parentheses.

### Hypothesis Testing

#### The Relationship Between Role Overload and Voice

To test Hypotheses 1a and 1b, we conducted an ordinary least squares regression. Employee perceived role overload at Time 1 was positively associated with self-reported withdrawal (β = 0.291, *p* < 0.001) and job crafting (β = 0.139, *p* < 0.05) at Time 2. To test for the mediation effect outlined in Hypotheses 2a and 2b, we used Model 4 of Hayes (2013) PROCESS macro for SPSS with 2,000 bootstrapped samples and included the control variables. The indirect effect is significant if the 95% confidence interval (CI) excludes zero. The analyses indicated a significant negative relationship between role overload and voice via withdrawal (indirect effect = −0.039, SE = 0.010, 95% CI = −0.061 to −0.022; direct effect = −0.009, SE = 0.026, 95% CI = −0.061 to 0.042) and a significant positive relationship between role overload and voice through job crafting (indirect effect = 0.017, SE = 0.007, 95% CI = 0.005 to 0.032; direct effect = −0.009, SE = 0.026, 95% CI = −0.061 to 0.042) (for an index summary, see Table 3). Together, these results provide support for Hypotheses 1a, 1b and 2a, 2b.

**TABLE 3:** Summary of indirect effects and moderated indirect effects.

Effects	Estimates	*SE*	95% confidence intervals
**Perceived role overload → withdrawal → Voice**			
Indirect effects	–0.039	0.010	[−0.061, −0.022]
Moderated mediation			
High-LMX	–0.030	0.011	[−0.053, −0.010]
Low-LMX	–0.047	0.013	[−0.075, −0.023]
**Perceived role overload → Job crafting → Voice**			
Indirect effects	0.017	0.007	[0.005, 0.032]
Moderated mediation			
High-LMX	0.042	0.011	[0.021, 0.065]
Low-LMX	0.011	0.010	[−0.007, 0.032]

#### Moderating Effect of Leader–Member Exchange

**FIGURE 2:**
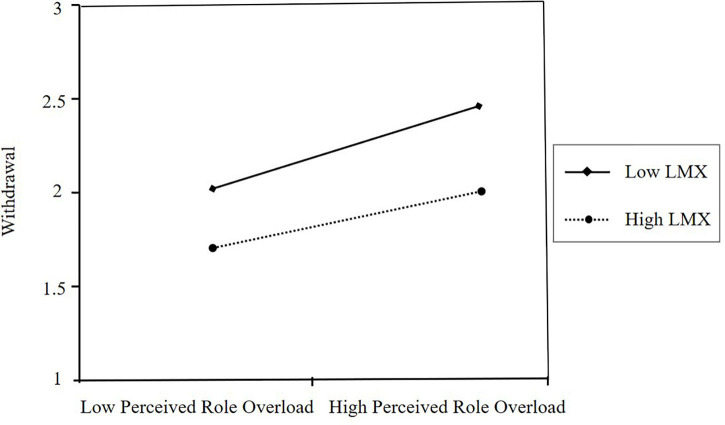
The interactive effect of perceived role overload and LMX on withdrawal.

To test the moderating effect of LMX, we examined the interactive effect of LMX and perceived role overload on withdrawal. As Table 4 illustrates, perceived role overload (β = 0.273, *p* < 0.001) was positively associated with withdrawal, whereas LMX (β = −0.251, *p* < 0.001) was negatively associated with withdrawal (Model 2 and 3). After the inclusion of the interaction term, the model did not explain more variance (*R*^2^ = 0.239; Δ*R*^2^ = 0.003, n.s.) and showed that the interaction term was not significant (β = −0.276, n.s.). We plotted the interaction (see Figure 2) to aid interpretation and found that it was not within our expectations. Then the methods of Hayes (2013) were used to test Hypothesis 3a. The results suggested that when LMX was low (one standard deviation below the mean of the LMX), the mediated model was significant (conditional indirect effect = −0.047, SE = 0.013, 95% CI = −0.075 to −0.023, not including 0). When LMX was high (one standard deviation above the mean of the LMX), the mediated model was also significant (conditional indirect effect = −0.030, SE = 0.011, 95% CI = −0.053 to −0.010, not including 0). However, the index of moderated mediation was not significant (index = 0.009, SE = 0.008, 95% CI = −0.007 to 0.026, including 0). In other words, as perceived role overload increases, employees are more inclined to withdraw, no matter the quality of LMX. Hence, Hypothesis 3a was not supported.

**TABLE 4:** Regression analyses for the effect of perceived role overload and withdrawal.

	Withdrawal			
	M1	M2	M3	M4
Age	−0.312***	−0.350***	−0.264**	−0.267**
Education	0.244***	0.184***	0.173***	0.174***
Income	–0.049	–0.048	–0.071	–0.073
Tenure	0.157	0.171*	0.147	0.155
Role overload		0.273***	0.241***	0.465**
LMX			−0.251***	–0.067
Role overload × LMX				–0.276
*R* ^2^	0.109	0.179	0.236	0.239
Δ*R*^2^		0.071	0.057	0.003
*F*	13.553***	19.415***	22.864***	19.878***
Δ*F*		38.315***	33.092***	1.735

n = 466; ***p < 0.001, **p < 0.01, *p < 0.05.

**FIGURE 3:**
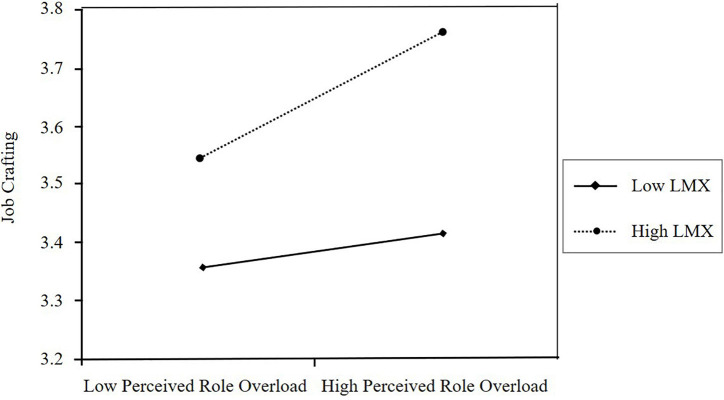
The interactive effect of perceived role overload and LMX on job crafting.

To test Hypothesis 3b, we followed the same procedure. First, we found that both perceived role overload (β = 0.118, *p* < 0.05) and LMX (β = 0.341, *p* < 0.001) are positively related to job crafting (Table 5, Model 6 and 7). The results show that after the incorporation of the interaction term, the model explained significantly more variance (*R*^2^ = 0.175; Δ*R*^2^ = 0.010, *p* < 0.05) and that the interaction term was significant (β = 0.516, *p* < 0.05). We plotted the interaction (see Figure 3) to aid interpretation; the figure indicated an expected direction. In addition, the methods of Hayes (2013) were used to test the moderated mediation effect. The results suggested that when LMX was low (one standard deviation below the mean), the mediated model was not significant (conditional indirect effect = 0.011, SE = 0.010, 95% CI = −0.007 to 0.032, including 0). When LMX was high (one standard deviation above the mean of the LMX), however, the mediated model was significant (conditional indirect effect = 0.042, SE = 0.011, 95% CI = 0.021 to 0.065, not including 0). The index of moderated mediation was significant (index = 0.017, SE = 0.008, 95% CI = 0.002 to 0.034, not including 0). This provides full support for Hypothesis 3b (for the index summary, see Table 3). In other words, although perceived role overload is generally associated with employees’ voice behavior, this effect is stronger when LMX is high but dissipated when LMX is low.

**TABLE 5:** Regression analyses for the effect of perceived role overload and job crafting.

	Job Crafting			
	M5	M6	M7	M8
Age	0.239**	0.223*	0.105	0.112
Education	0.082	0.056	0.071	0.070
Income	–0.050	–0.049	–0.018	–0.014
Tenure	–0.014	–0.008	0.024	0.010
Role overload		0.118*	0.162***	–0.256
LMX			0.341***	–0.002
Role overload × LMX				0.516*
*R* ^2^	0.046	0.059	0.165	0.175
Δ*R*^2^		0.013	0.105	0.010
*F*	5.399***	5.616***	14.583***	13.426***
Δ*F*		6.23*	55.942***	5.581*

n = 466; ***p < 0.001, **p < 0.01, *p < 0.05.

## Discussion

Through a two-wave field study in China, we found support for our hypotheses that role overload is a mixing stressor. Building on the hindrance–challenge framework (Cavanaugh et al., 2000) and the BIS/BAS perspective (Carver, 2006), our study showed that, on the one hand, role overload positively related to a typical BIS response (withdrawal), which in turn is negatively associated with employees’ extra-role performance (voice). On the other hand, role overload also positively correlated with a typical BAS response (job crafting), which in turn is positively associated with extra-role performance (voice). We further demonstrate that the positive mediated effect was moderated by LMX. Specifically, our findings indicate that the higher the LMX relationship is rated by employees, the stronger the positive relationship between role overload and the BAS response (job crafting). However, inconsistently with our expectations that LMX would buffer the positive association between role overload and the BIS response (withdrawal), our findings indicate that the relationship between role overload and withdrawal remains positive, no matter the quality of LMX. One possible explanation is that humans are more sensitive to losses and threats (Hobfoll, 2001). Even though a good LMX relationship with the supervisor implies for employees that role overload contains an opportunity for gaining reward and personal growth, thus inducing employees to display proactive behaviors (job crafting), people may not let down their guard against the potential threats of role overload. This is not contradictory because, according to theoretical and empirical findings, the BIS and the BAS are two independent systems (Carver, 2006; Sherf et al., 2021); therefore, withdrawal and job crafting are independent behaviors such that employees can proactively pursue rewards and growth when the LMX relationship is better while simultaneously displaying withdrawal behaviors to keep threat situations away. For example, A kindergarten teacher who has a high-LMX quality with a superior may try her/his best to organize the Children’s Day party while completing her/his daily teaching tasks (a situation that may lead to role overload), but she or he may also try to keep her/his distance from redundant tasks to avoid exhaustion. That is, while a better LMX relationship could make employees work harder to complete challenging tasks, it may not prevent them from avoiding obstructive tasks. Indeed, prior studies have suggested that threat and challenge appraisals are not mutually exclusive and can occur simultaneously (Folkman, 1984).

### Theoretical Implications

By expanding upon and complementing the previous empirical findings in the role stressors literature, this paper makes some important contributions. First, our findings have contributed to the literature by proposing and testing an integrated model through which role overload can be experienced as hindrance and challenge stressors simultaneously, thus being in turn negatively and positively related to extra-role performance. In doing so, the current model helps reconcile the disparate findings pertaining to the effects of role overload and extra-role performance, as previous scholars have mentioned (Eatough et al., 2011). In fact, we are not the first to argue for the duality of role overload; others have noted that role overload contains components both of hindrance and challenge stressors (Gilboa et al., 2008; Eatough et al., 2011). The present results, however, provide the first empirical evidence for the hypothesis that employees may perceive role overload as a hindrance and as a challenge stressor. For future research about role stressors, our results suggest that we should pay more attention to the duality of role overload. Our understanding may not be comprehensive if we just regard role overload as either a hindrance or a challenge stressor.

Second, our study also contributes to the BIS/BAS framework. Our results indicate that the relationships between role overload and extra-role performance are channeled through withdrawal and job crafting, indirectly suggesting that the BIS and BAS regulation systems (Carver, 2006) may play important roles in these relationships. By arguing that withdrawal and job crafting may represent two prototypical responses of two independent and biologically based self-regulation systems, the BIS and BAS, this study helps to expand the application of the BIS/BAS perspective to explain the controversial findings regarding role overload (Eatough et al., 2011; Pooja et al., 2016; Montani and Dagenais-Desmarais, 2018). This method is consistent with the study by Sherf et al. (2021), which drew on the BIS/BAS perspective to distinguish between two distinct behavioral responses to generate theory for distinct antecedents and outcomes.

Third, our study revealed the moderating effects of LMX, thus extending the booming literature on leadership and job-related stressors. Prior studies have uncovered the important role of leadership and LMX in the relationship between role stressors and workplace outcomes. For instance, a high level of social support from a supervisor can weaken the negative influence of role stressors on deviant behavior by employees (Chiu et al., 2015). Authentic leadership can influence subordinates’ OCBs through role overload (Zhang and Xie, 2017). Leaders’ need for structure can moderate the association between role overload and job crafting, such that job crafting is strongest when leaders’ need for structure is low (Solberg and Wong, 2016). However, the results regarding the role of LMX have not always been consistent with researchers’ expectations. For example, some studies have demonstrated that LMX can reduce the negative influence of hindrance stressors on task performance and OCBs (McLarty et al., 2020), as well as affective commitment (Montani et al., 2017). Nevertheless, contrary to the expectation that LMX acts as a buffer against the negative correlation between stressors and ego depletion, Xia et al. (2020) found that hindrance stressors are associated with higher resource depletion in employees with high-LMX relationships. Thus, our study offers a new understanding of how LMX can impact the process for employees to cope with job stressors. That is, higher LMX quality may enhance the positive effects but difficult to buffer the negative effects of role overload on extra-role performance.

### Practical Implications

Our findings have several important implications for practice. The broader implication of our study is that employees and supervisors need to be mindful that role overload, one of the most common role stressors in the workplace, is a stressor containing both hindrance and challenge components. Thus, viewing role overload dialectically and making a greater attempt to maximize the challenging facet and minimize the hindrance facet of role overload is crucial.

For employees, it should be beneficial to recognize the growth and reward opportunities role overload may contain. When employees perceive themselves as having too many responsibilities or activities, the best coping strategy is not to exhibit withdrawal behavior (e.g., absenteeism, reduce effort), as this is harmful to their performance, and a low performance level may have a negative impact on their career development and promotion prospects. It could be beneficial to cope with role overload proactively.

For organizations, the negative effects of role overload should be of particular concern. Our study indicated that the negative effects of role overload on performance may be difficult to eliminate. Unfortunately, the relative costs and benefits of role overload are still poorly understood. However, effective management of role overload is undoubtedly beneficial. Organizations should provide support to employees. This could be reducing someone’s workload to just what is necessary (American Psychological Association, 2019). Moreover, as we have noted at the beginning, technology (e.g., instant message) may increase employees’ perception of role overload. Some practices that help workers recover from exhaustion may be beneficial, such as arranging a smartphone-free night for employees (Colbert et al., 2016), and human resources practices like internal marketing that is helpful to ease the organizational stress (Nemteanu and Dabija, 2021).

Finally, according to our research findings, leaders play a key role in influencing BIS/BAS responses to role overload. Supervisors should bear in mind the key role they may play in the process of subordinates’ interpretation of role overload. Thus, we suggest that it can be beneficial for supervisors to improve their leadership skills, especially those that could help them transmit key information about reward prospects and encourage subordinates to take responsibility proactively (Piccolo and Colquitt, 2006). Moreover, both supervisors and subordinates should try their best to develop higher LMX quality to evoke better performance from employees (Montani et al., 2017).

### Limitations and Future Research

Our study has some limitations that bear mentioning. First, as with most organizational research, our research employed a non-experimental design, which prevents us from making causal inferences regarding the linkages in the model. To fully pinpoint causality, an experimental design would be better suited, so as to rule out other possible alternative pathways. Second, the measurement of study variables relied on employees’ self-reports. Therefore, our study is not free from common method biases (Podsakoff et al., 2003). However, we used a time-lagged design, which may reduce concerns about this limitation. Third, the analysis unit of our research is between-individual, so it cannot find out more precise information about the outcomes of role overload. Long-term longitudinal studies are needed that can provide a more accurate understanding of within-person changes in response to role overload. Indeed, as argued by Sherf et al. (2021), the BIS and BAS are different and independent regulation systems; thus, the prototypical responses of BIS and BAS can appear simultaneously. Thus, it is possible for an employee to exhibit BIS and BAS responses in a single day. It would be interesting to extend the theory regarding how within-person variance in role overload predicts changes in BIS and BAS responses over time. Finally, we relied on BIS/BAS perspective accounts for the mediating role of role overload’s effects but did not include variables representing the process through which such effects are transmitted. Instead, consistent with a prior study (Sherf et al., 2021), we argued for the two prototypical behaviors of BIS/BAS as mechanisms between role overload and extra-role performance. This is because current measurements of BIS/BAS are used in the research area of personality psychology (Carver and White, 1994; Diefendorff and Mehta, 2007). It may be helpful in the future to develop a measure of the two dimensions to capture the state of BIS/BAS regulations, as in other approach/avoidance self-regulatory studies (Neubert et al., 2008).

## Conclusion

Taken together, the current research helps uncover the mechanisms and boundary conditions associated with the effects of role overload on employees’ extra-role performance. Drawing on an integrated perspective of the hindrance–challenge framework as well as the BIS/BAS perspective, we break new ground in research on role overload. Our findings examined the idea that role overload contains both hindrance and challenge components by offering empirical evidence that role overload can activate two prototypical behavioral responses of the BIS and BAS regulatory systems—withdrawal and job crafting —which, in turn, exert negative and positive effects, respectively, on extra-role performance. Moreover, our results indicated that when LMX is high, the negative effect of role overload still existed; the positive effect of role overload, however, is boosted. In sum, our research has revealed the duality characteristic of role overload and has clarified how role overload affects extra-role performance, taking us one step further toward a thorough understanding of the relationship between role overload and extra-role performance.

## Data Availability Statement

The raw data supporting the conclusions of this article will be made available by the authors, without undue reservation.

## Ethics Statement

The studies involving human participants were reviewed and approved by Ethics Committee of Sichuan Normal University. The patients/participants provided their written informed consent to participate in this study.

## Author Contributions

BH contributed to the central idea. BH and LM analyzed most of the data and wrote the initial draft of the manuscript. WX collected part of the data, carried out additional analyses, and finalized the manuscript. All authors discussed the results and revised the manuscript.

## Conflict of Interest

The authors declare that the research was conducted in the absence of any commercial or financial relationships that could be construed as a potential conflict of interest.

## Publisher’s Note

All claims expressed in this article are solely those of the authors and do not necessarily represent those of their affiliated organizations, or those of the publisher, the editors and the reviewers. Any product that may be evaluated in this article, or claim that may be made by its manufacturer, is not guaranteed or endorsed by the publisher.
